# Sudden infant death syndrome: a re-examination of temporal trends

**DOI:** 10.1186/1471-2393-12-59

**Published:** 2012-06-29

**Authors:** Sarka Lisonkova, Jennifer A Hutcheon, KS Joseph

**Affiliations:** 1 Department of Obstetrics & Gynaecology, University of British Columbia and the Women’s Hospital and Health Centre of British Columbia, Room E418B, 4480 Oak Street, Vancouver, BC, V6H 3 V4, Canada; 2 School of Population and Public Health, University of British Columbia, Vancouver, Canada

**Keywords:** SIDS, Temporal trend, Gestational age

## Abstract

**Background:**

While the reduction in infants’ prone sleeping has led to a temporal decline in Sudden Infant Death Syndrome (SIDS), some aspects of this trend remain unexplained. We assessed whether changes in the gestational age distribution of births also contributed to the temporal reduction in SIDS.

**Methods:**

SIDS patterns among singleton and twin births in the United States were analysed in 1995–96 and 2004–05. The temporal reduction in SIDS was partitioned using the Kitagawa decomposition method into reductions due to changes in the gestational age distribution and reductions due to changes in gestational age-specific SIDS rates. Both the traditional and the fetuses-at-risk models were used.

**Results:**

SIDS rates declined with increasing gestation under the traditional perinatal model. Rates were higher at early gestation among singletons compared with twins, while the reverse was true at later gestation. Under the fetuses-at-risk model, SIDS rates increased with increasing gestation and twins had higher rates of SIDS than singletons at all gestational ages. Between 1995–96 and 2004–05, SIDS declined from 8.3 to 5.6 per 10,000 live births among singletons and from 14.2 to 10.6 per 10,000 live births among twins. Decomposition using the traditional model showed that the SIDS reduction among singletons and twins was entirely due to changes in the gestational age-specific SIDS rate. The fetuses-at-risk model attributed 45% of the SIDS reduction to changes in the gestational age distribution and 55% of the reduction to changes in gestational age-specific SIDS rates among singletons; among twins these proportions were 64% and 36%, respectively.

**Conclusion:**

Changes in the gestational age distribution may have contributed to the recent temporal reduction in SIDS.

## Background

Although Sudden Infant Death Syndrome (SIDS) is a leading cause of post-neonatal death in industrialized countries, its etiology is largely unknown [1]. While the reduction in prone sleeping following the back-to-sleep campaign has led to a decline in SIDS in many countries [2-6], there are several puzzling aspects related to this intervention and the epidemiology of SIDS. For instance, the onset of the decline in SIDS preceded the initiation of the back-to-sleep campaign [2-7]. The reduction in SIDS in the United States began in 1989, while the back-to-sleep campaign was initiated in 1994 [6]. Similarly, SIDS rates in the United Kingdom decreased continuously from 1988 onwards, while the back-to-sleep campaign only began in 1991 [7].

Other unexplained epidemiologic features of the temporal reduction in SIDS include the relatively greater reduction in SIDS among term infants, as compared with infants born at preterm gestation. Data from Avon county in England show that term live births among SIDS cases decreased from 88% in 1984–88 to 63% in 1994–98, the period when SIDS rates declined most rapidly. The proportion of term infants among SIDS cases remained stable thereafter (66% in 1999–2003) and SIDS rates did not change dramatically during this period [7]. Also, a larger decline in SIDS was observed among twins as compared with singletons. In England, SIDS among twin live births declined by 71% from 1.4 per 1000 live births in 1993 to 0.4 per 1000 live births in 2003, whereas among singletons, SIDS rates decreased by 50% from 0.6 to 0.3 per 1000 live births during the same period [8,9]. It is notable that births at term and post-term gestation and twin births (subpopulations which experienced relatively larger reductions in SIDS) also experienced the largest increases in early delivery (i.e., increased obstetric intervention through labour induction and cesarean delivery).

Perhaps the most intriguing finding related to SIDS is the paradoxical association between plurality and birth weight-specific SIDS rates [8,9]. SIDS rates are higher among twins as compared with singletons among normal birth weight infants (>3,000 g), whereas at lower birth weights the opposite is true. This phenomenon, sometimes referred to as the paradox of intersecting mortality curves, has also been observed when birth weight- and gestational age-specific stillbirth or infant mortality rates are contrasted across plurality, parity, race and other factors [10].

Various explanations [11] have been proposed to explain the paradox of intersecting mortality curves including the fetuses-at-risk approach [12,13]. This model assumes an intrauterine etiology for the outcomes of interest and gestational age-specific mortality rates calculated using the fetuses-at-risk approach do not exhibit the crossover paradox [14]. Since numerous studies have shown that unexplained antepartum fetal death and SIDS have common features (including similar pathologic characteristics at autopsy [15-17], common risk factors [15,18,19] and congruent temporal trends [1-7,20]), there is good justification for using the fetuses-at-risk approach for examining gestational age-specific SIDS rates. Finally, the contrast between the gestational age-specific patterns of SIDS and diseases of prematurity (e.g., retinopathy of prematurity) suggest that SIDS is a late gestation disease whose incidence may have been affected by temporal changes in the gestational age distribution [14].

In this study we explored the extent to which changes in gestational age distribution and changes in gestational age-specific SIDS rates contributed to the temporal decline in SIDS among singletons and twins.

## Methods

We used population-based data on singleton and twin births in the United States from 1995–96 to 2004–05 from the National Centre for Health Statistics (NCHS). Information in the NCHS birth/death and fetal death files was abstracted from birth certificates and is publicly available [21], with the birth-infant death linkage carried out by the NCHS (period linked birth-infant death file). We included all infants born at ≥22 week gestation, based on the clinical estimate of gestation at birth [22-24]. States that did not report the clinical estimate of gestational age were not included in the analysis (13.5% of all births). We excluded infants weighing less than 500 grams in order to avoid potential bias due to variable birth registration at the borderline of viability [25-27]. International Classification of Diseases (ICD) codes 798.0 and R95 (ICD 9^th^ version, and ICD 10^th^ version, respectively) were used to identify cases of SIDS. Information about maternal and infant risk factors associated with SIDS, including maternal age, education, race, parity, and marital status, was also obtained from the NCHS files. Temporal changes in the frequency of these risk factors were evaluated by contrasting their population prevalence between 1995–96 and 2004–05. Temporal changes in SIDS rates across categories of each risk factor were evaluated using rate ratios (2004–05 vs. 1995–96) and 95% confidence intervals.

Gestational age-specific SIDS rates were compared using two different approaches: A) the traditional method which expressed gestational age-specific SIDS rates as the number of SIDS cases at any gestation divided by the number of live births at that gestation; and B) the fetuses-at-risk approach. Under the fetuses-at-risk approach, gestational age-specific SIDS rates were calculated as the number of SIDS cases among infants born at any gestation divided by the number of fetuses in-utero who were at risk of birth (live birth or stillbirth) at that gestation [12,13]. This latter model assumes an intrauterine etiology for SIDS and has been used previously for estimating gestational age-specific rates of stillbirth, neonatal death and cerebral palsy [12,13]. Both approaches were used to examine the temporal trends in SIDS, because they embody different perspectives; the traditional approach models the gestational age-specific risk of SIDS after birth assuming that live births are the appropriate candidates for SIDS, whereas the fetuses-at-risk approach models an in-utero etiology and assumes that fetuses are the appropriate candidates for SIDS.

The temporal trend in SIDS was conceptualized as a potential consequence of temporal changes in the gestational age distribution and/or as a consequence of temporal changes in the gestational age-specific SIDS rates (e.g., due to the back to sleep campaign). The relative contribution of each of these two components to the overall reduction in SIDS was estimated by the Kitagawa decomposition method [28]. This method partitions the mortality rate difference between the two time periods into two components: the mortality difference due to the change in the gestational age distribution and the mortality difference due to the change in gestational age-specific mortality. By holding one component constant at its average (e.g., average gestational age–specific SIDS rate), the Kitagawa method estimates the relative contribution of the second component (i.e., the gestational age distribution), and vice versa. The Kitagawa decomposition formula is expressed as:

(1) $$\text{N1-N}2=\sum_{i=1}^{n}\frac{\left(R_{1i}+R_{2i}\right)}{2}\left(F_{1i}-F_{2i}\right)+\sum_{i=1}^{n}\frac{\left(F_{1i}+F_{2i}\right)}{2}\left(R_{1i}-R_{2i}\right)$$

where N1 and N2 denote SIDS rates in 2004–05 and 1995–96, respectively, R_1_ and R_2_ refer to gestational age-specific SIDS rates in 2004–05 and 1995–96, F_1_ and F_2_ represent proportions of live births in gestational age category *i* for each respective time period, and *i* denotes gestational age category (in weeks). The first part of the equation represents the relative contribution of changes in the gestational age distribution to the overall difference in SIDS rates, and the latter part of the equation represents the relative contribution of changes in gestational age-specific SIDS rates. For the decomposition using the traditional approach, the gestational age distribution at gestational week *i* was defined as the number of live births at that gestation expressed as a proportion of all live births; for the decomposition using the fetuses-at-risk approach, the gestational age distribution at gestational week *i* was expressed as the number of live births at that gestation expressed as a fraction of all fetuses in-utero at that gestation.

The Kitagawa decomposition was carried out separately for singletons and twins born at term vs pre-term gestation (≥37 weeks vs 22–36 weeks).

New birth certificates were introduced in the United States in 2003 and led to some increases in missing values for a few variables of interest (e.g., educational status, congenital malformations). Sensitivity analyses were carried out to assess how these changes affected results by restricting temporal trends to a period before the introduction of the new birth certificate i.e., between 1995–96 and 2001–02. All analyses were carried out using SAS version 9.2. Data used in this study were publicly accessible from the National Centre for Health Statistics [21].

## Results

The rate of SIDS declined from 8.3 to 5.6 per 10,000 live births from 1995–96 to 2004–05 among singletons (rate difference −2.7, 95% CI: -2.4 and −3.0), and from 14.2 to 10.6 per 10,000 live births among twins (rate difference −3.6, 95% CI: -1.4 and −5.9). On a relative scale SIDS rates declined by 33% (rate ratio 0.67, 95% CI: 0.67-0.67) among singletons and by 25% among twins (rate ratio 0.75, 95% CI: 0.74-0.75).

Changes in maternal characteristics between 1995–96 and 2004–05 are shown in Table 1. The proportion of older, Hispanic and unmarried mothers increased, while the proportion of mothers who were less than 20 years old, non-Hispanic white, and those who smoked during pregnancy decreased from 1995–96 to 2004–05. The frequency of twin live births increased, while gestational age at delivery decreased. There was a increase in the proportion of live births at preterm gestation (from 8.6% to 10.6% at <37 weeks) and at early term gestation (from 21.2% to 29.3% at 37–38 weeks) and a decrease in the proportion of live births at late term gestation (from 67.8% to 59.6% at 39–41 weeks) and post-term gestation (from 2.3% to 0.7% at ≥42 weeks). SIDS rates declined during this period across all maternal characteristics (Table 1).

**Table 1:** Changes in maternal and infant characteristics and SIDS rates, United States, 1995–96 and 2004-2005

**Maternal/infant characteristics**	**1995-96**				**2004-05**				**Rate ratio**
	**Live births**		**SIDS**		**Live births**		**SIDS**		
	**N**	**%**	**N**	**Rate per 1000 live births**	**N**	**%**	**N**	**Rate per 1000 live births**	
Age (years) <20	868,673	13.2	1,429	1.65	736,649	10.4	792	1.08	0.65 (0.60-0.71)
20-24	1,627,849	24.6	2,035	1.25	1,812,729	25.5	1,741	0.96	0.77 (0.72-0.82)
25-29	1815,432	27.5	1,123	0.62	1,936,846	27.3	901	0.47	0.75 (0.69-0.82)
30-34	1,527,046	23.1	685	0.45	1,634,468	23.0	415	0.25	0.57 (0.50-0.64)
35-39	650,020	9.8	256	0.39	800,732	11.3	189	0.24	0.60 (0.50-0.72)
40+	115,534	1.7	45	0.39	180,408	2.5	39	0.22	0.56 (0.36-0.85)
Race: Non-Hispanic white	4,307,044	65.2	3,152	0.73	4,223,756	59.5	2,323	0.55	0.75 (0.71-0.79)
African American	1,071,246	16.2	1,690	1.58	1,089,924	15.3	1,150	1.06	0.67 (0.62-0.72)
Hispanic	857,735	13.0	452	0.53	1,364,849	19.2	426	0.31	0.59 (0.52-0.68)
Other/unknown	368,529	5.6	134	0.36	423,303	6.0	83	0.20	0.54 (0.41-0.71)
Education* < high school	1,342,264	20.3	2262	1.69	1,445,734	20.4	1,468	1.02	0.60 (0.56-0.64)
Smoking during pregnancy	824,813	12.5	1894	2.30	707,215	10.0	1,372	1.94	0.84 (0.79-0.91)
Single parent	2,132,386	32.3	3235	1.52	2,592,271	36.5	2,561	0.99	0.65 (0.62-0.69)
No prior live births	2,742,409	41.5	1644	0.60	2,825,916	39.8	1,140	0.40	0.67 (0.62-0.73)
Infant sex (male)	3,379,795	51.2	3294	0.97	3,636,621	51.2	2,466	0.68	0.70 (0.66-0.73)
Singleton infants	6,436,128	97.4	5333	0.83	6,872,585	96.8	3,833	0.56	0.67 (0.65-0.70)
Congenital anomalies*	101,446	1.5	94	0.93	78,727	1.1	44	0.56	0.60 (0.42-0.86)
Gestational age (weeks):									
22-36	573,469	8.68	1,138	1.98	746,046	10.50	918	1.23	0.62 (0.57-0.68)
≥37	6,031,085	91.32	4,435	0.74	6,355,786	89.50	3,159	0.50	0.68 (0.65-0.71)
Total	6,604,554	100.00	5,573	0.84	7,101,832	100.0	4077	0.57	0.68 (0.65-0.71)

(Figure 1a shows the gestational age-specific rates of SIDS among singleton and twin live births between 28 and 40 weeks gestation as calculated under the traditional perinatal model. Rates of SIDS declined with increasing gestational age among both singletons and twins. Rates of SIDS were lower among twins at preterm gestation compared with singleton live births at preterm gestation, but the opposite was true at later gestational ages (paradox of intersecting perinatal mortality curves). (Figure 1b shows gestational age-specific rates of SIDS among singletons and twins under the fetuses-at-risk model. Rates of SIDS increased with increasing gestation among both singletons and twins and SIDS rates were higher among twins at all gestational ages.

**Figure 1:**
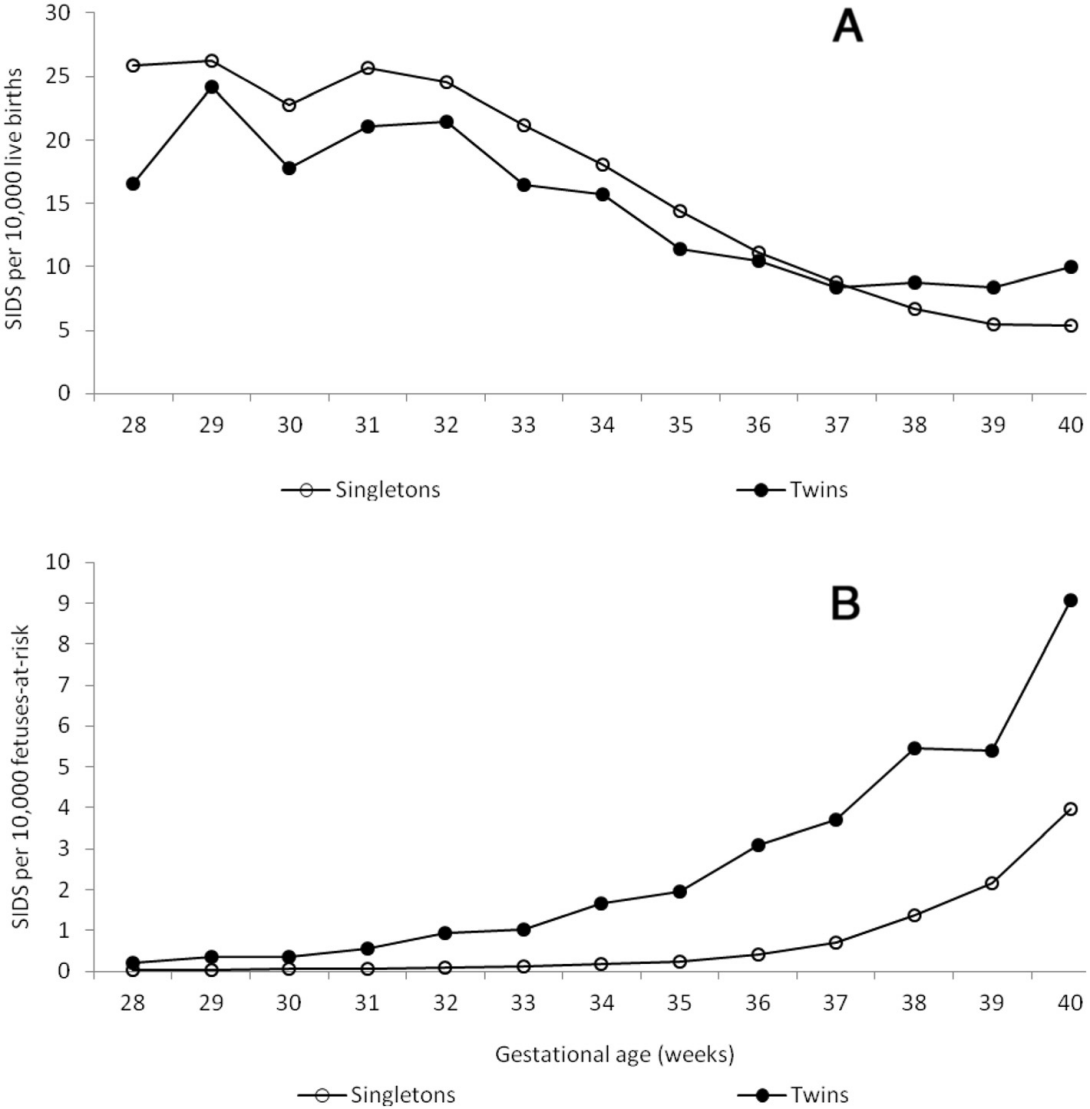
**Gestational age-specific rates of SIDS between 28 and 40 weeks gestation among singleton and twin live births according to the traditional perinatal model (Figure**1**a) and according to the fetuses-at-risk model (Figure**1**b), United States, 1995–2005**.

**Figure 2:**
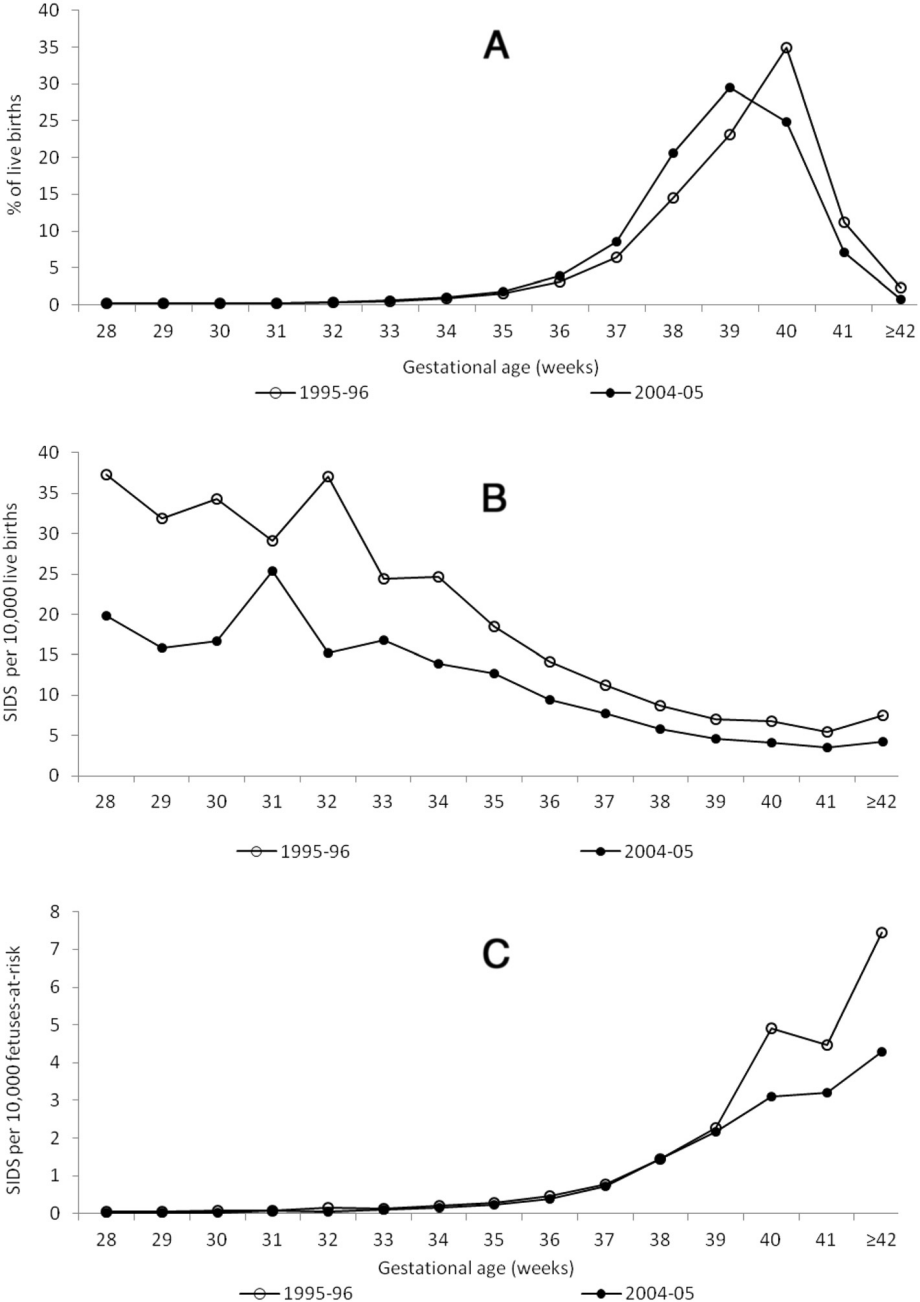
**Changes in the gestational age distribution of singleton live births (Figure**2**a), in gestational age-specific rates of SIDS (traditional model, Figure**2**b) and in gestational age-specific rates of SIDS (fetuses-at-risk model, Figure**2**c) among singletons 28 to 40 weeks gestation, United States, 1995–96 and 2004–05**.

**Figure 3:**
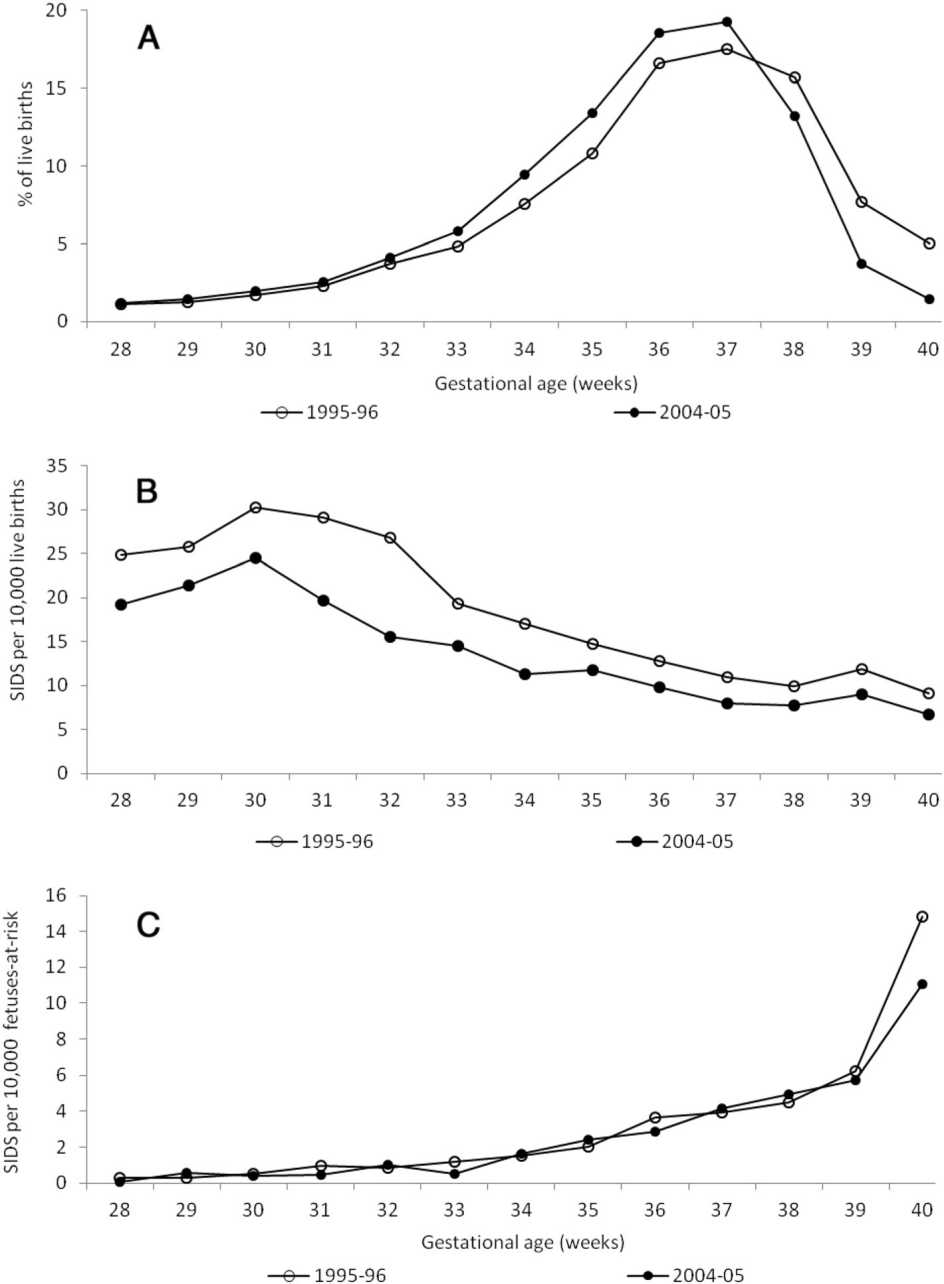
**Changes in the gestational age distribution of twin live births (Figure**3**a), in gestational age-specific rates of SIDS (traditional model, moving average, Figure**3**b,) and in gestational age-specific rates of SIDS (fetuses-at-risk model, Figure**3**c) among twins between 28 and 40 weeks gestation, United States, 1995–96 and 2004–05**

Substantial changes occurred in the gestational age distribution of singleton live births between 1995–96 and 2004–05 ((Figure 2a). The proportion of singleton live births at gestational ages up to 39 weeks increased, while the proportion after 39 weeks declined. Under the traditional model, gestational age-specific SIDS rates showed a temporal decline at all gestational ages (Figure 2b), while under the fetuses-at-risk approach, gestational age-specific SIDS rates showed a temporal decline at 39 weeks and later (Figure 2c). (Figure 3a shows changes in the gestational age distribution among twins between 1995-96 and 2004–05; the proportion of live births up to 37 weeks increased and there was a decline in the proportion of births at 38 weeks and later. Gestational age-specific SIDS rates among twins showed a temporal decline at all gestations under the traditional model ((Figure 3b), while under the fetuses-at-risk model, no decline in rates was evident except at 40 weeks of gestation (Figure 3c).

Table 2 presents rates of SIDS among singletons and twins at preterm and term gestation in 1995–96 and in 2004–05, with temporal changes expressed in terms of rate differences and rate ratios. Under the traditional model, singletons showed a larger relative decline in SIDS than twins (rate ratio 0.67 vs 0.75), whereas in absolute terms twins showed a larger reduction than singletons (rate difference −3.61 vs −2.72 per 10,000 live births). Reductions in SIDS rates were larger at preterm gestation compared with term gestation in terms of both the ratio and the difference measure. Under the fetuses-at-risk approach, temporal changes were larger at term gestation among both singletons and twins irrespective of the effect measure (whether ratio or difference).

**Table 2:** SIDS rates, rate ratios and rate differences, by plurality and gestation, United States, 2004-05 vs. 1995–96

**Plurality and gestational age (weeks)**	**Traditional approach**				**Fetuses-at-risk approach**			
	**1995-96**	**2004-05**	**2004-05 vs 1995-96**		**1995-96**	**2004-05**	**2004-05 vs 1995-96**	
	**SIDS rate (per 10,000 live births)**	**SIDS rate (per 10,000 live births)**	**Rate difference**	**Rate ratio (95% CI)**	**SIDS rate (per 10,000 FAR)**	**SIDS rate (per 10,000 FAR)**	**Rate difference**	**Rate ratio (95% CI)**
Singletons								
22-36 weeks	20.34	12.31	−8.0	0.61 (0.60-0.61)	1.52	1.08	−0.4	0.71 (0.71-0.71)
≥37 weeks	7.32	4.93	−2.4	0.67 (0.67-0.67)	6.74	4.48	−2.3	0.67 (0.67-0.67)
Total	8.30	5.58	−2.7	0.67 (0.67-0.67)	8.26	5.56	−2.7	0.67 (0.67-0.67)
Twins								
22-36 weeks	17.65	12.27	−5.4	0.70 (0.69-0.70)	9.35	7.59	−1.8	0.81 (0.81-0.82)
≥37 weeks	10.34	7.96	−2.4	0.77 (0.76-0.78)	10.32	7.95	−2.3	0.77 (0.76-0.78)
Total	14.25	10.64	−3.6	0.75 (0.74-0.75)	14.12	10.58	−3.5	0.75 (0.74-0.75)

95% CI denotes 95% confidence intervals; SIDS cases were defined by the underlying cause of death 7980 (ICD-9) in 1995–06 and R95 (ICD-10) in 2004–05. The discrepancy in total SIDS rates under the traditional and fetuses-at-risk approaches is because stillbirths were included in the denominator for the latter calculation. Rate differences were calculated per 10,000 live births and 10,000 fetuses-at-risk.

Among singletons, the traditional Kitagawa decomposition method revealed that the overall temporal reduction in SIDS rates (−2.7 cases per 10,000 live births) was entirely due to the decrease in gestational age-specific SIDS rates (Table 3). In fact, under this model, changes in the gestational age distribution adversely impacted SIDS rates. However, the modified Kitagawa decomposition method, based on the fetuses-at-risk approach, yielded a different partitioning. Changes in the gestational age-specific distribution were responsible for 45% of the overall decline in SIDS (−1.2 cases per 10,000 fetuses-at-risk), whereas changes in gestational age-specific rates were responsible for 55% of the overall decline (−1.5 cases per 10,000 fetuses-at-risk, Table 3)..

**Table 3:** Relative contribution of changes in the gestational age distribution and in gestational age-specific SIDS rates to the overall reduction in SIDS rates by plurality and gestation, United States, 2004-05 vs. 1995–96

**Kitagawa decomposition – traditional method (per 10,000 live births)**						
**Plurality and gestational age (weeks)**	**Contribution of changes in gestational age distribution**	**Contribution of changes in gestational age-specific SIDS rate**	**Sum of both components (overall decline)**	**Relative contribution of changes in gestational age distribution (%)**	**Relative contribution of changes in gestational age- specific SIDS rate (%)**	**Sum of both components (%)**
Singletons						
22-36 weeks	+0.18	−0.63	−0.45	+40.0	−140.0	100
≥37 weeks	+0.19	−2.46	−2.27	+8.4	−108.4	100
Total	+0.37	−3.09	−2.72	+13.6	−113.6	100
Twins						
22-36 weeks	+1.29	−3.09	−1.81	+71.3	−171.3	100
≥37 weeks	−0.98	−0.82	−1.80	−54.3	−45.7	100
Total	+0.31	−3.92	−3.61	+8.6	−108.6	100
**Modified Kitagawa decomposition (per 10,000 fetuses-at-risk)**						
Singletons						
22-36 weeks	<0.01	−0.44	−0.44	<0.0	−100.0	100
≥37 weeks	−1.22	−1.04	−2.26	−54.0	−46.0	100
Total	−1.22	−1.48	−2.70	−45.2	−54.8	100
Twins						
22-36 weeks	−0.35	−1.42	−1.77	−19.7	−80.3	100
≥37 weeks	−1.90	+0.13	−1.77	−107.2	+7.2	100
Total	−2.25	−1.29	−3.54	−63.5	−36.5	100

Explanatory note: Between 1995–96 and 2004–05, singletons experienced a decrease in SIDS (−2.72 SIDS per 10,000 live births; 100% decrease). Under the traditional model, changes in gestational age distribution among singletons increased SIDS rates (+0.37 cases per 10,000 live births; 13.6% increase), whereas changes in gestational age-specific SIDS rates decreased SIDS rates (−3.09 cases per 10,000 live births; 113.6% decrease)

The decline in SIDS among twins followed a similar pattern. Under the traditional Kitagawa decomposition, the entire temporal change in SIDS rates (−3.6 cases per 10,000 live births) was due to changes in gestational age-specific mortality (Table 3). Under the fetuses-at-risk approach, however, the temporal shift in the gestational age distribution was responsible for 63% of the decline in SIDS (−2.3 cases per 10,000 fetuses-at-risk), while change in gestational age-specific SIDS rates were responsible for 37% of the observed SIDS reduction (−1.3 SIDS cases per 10,000 fetuses-at-risk, Table 3).

The decomposition of the SIDS decline yielded different results at preterm vs term gestation. Under the traditional model, the change in the gestational age distribution had a relatively larger adverse effect at preterm gestation among singletons, whereas among twins, the change in the gestational age distribution adversely affected SIDS rates at preterm gestation only. Changes in gestational age-specific SIDS rates were responsible for a larger proportion of the SIDS decline at preterm gestation compared to term gestation among both singletons and twins (Table 3). Under the fetuses-at-risk approach, gestational age distribution changes contributed substantially to the SIDS decline at term gestation among both singletons and twins. On the other hand, changes in gestational age-specific SIDS rates contributed more to the SIDS decline at preterm gestation compared to term gestation (Table 3).

Results from sensitivity analyses restricted to years prior to the introduction of new birth certificates in 2003 showed that between 1995–96 and 2001–02, the decline in SIDS rates was similar among singletons and twins. SIDS rates declined from 8.3 to 5.9 per 10,000 live births among singletons and from 14.2 to 10.5 per 10,000 live births among twins (rate ratio for singletons 0.72, 95% CI 0.69-0.75 and for twins 0.74, 95% CI 0.62-0.89).

## Discussion

Our study confirms the paradoxical relationship between plurality and SIDS under the traditional perinatal model; twins had lower rates of SIDS at preterm gestation, while singletons had lower rates at later gestation. The fetuses-at-risk approach eliminated the paradoxical crossover in SIDS rates by plurality and showed that, in fact, twins had higher rates of SIDS at all gestational ages. Analyses using the fetuses-at-risk approach also showed that temporal changes in the gestational age distribution of live births were responsible for 45% of the temporal decline in SIDS rates among singletons and for 64% of the decline among twins. The remainder of the decline in SIDS (55% among singletons and 37% among twins) was attributed to reductions in gestational age-specific rates of SIDS (e.g., due to the back to sleep campaign, etc.). This effect of a temporal shift in gestational age distribution was evident predominantly among term singletons and term twins suggesting that the temporal reduction in SIDS occurred, in part, due to a shift in the gestational age distribution among term infants.

While the exact cause of SIDS is unknown, the ‘triple risk hypothesis’ implicates three causes in the etiology of SIDS, namely, genetic factors, a critical developmental period, and environmental factors [29]. These three risk factors may affect the fetus/infant sequentially at different stages of development, and progressively increase the risk of SIDS. Genetic predisposition is related to male gender and race [29,30] and several gene polymorphisms involved in autonomic function, neurotransmission, energy metabolism, and response to infection have been implicated [31,32]. However, temporal changes in genetic factors are unlikely to explain the temporal decline in SIDS.

Critical developmental periods may occur at various time-windows during fetal or infant development. Suboptimal conditions in-utero, due to hypoxia, have been implicated in the origins of SIDS [15]. Studies have shown that infants who died of SIDS had a higher incidence of subcortical leukomalacia, brainstem gliosis, and other changes in central nervous system, when compared with infants not affected by SIDS [33]. These lesions have been found to originate, in part, in the antenatal period, suggesting that an antepartum hypoxic insult may constitute a predisposing risk factor for SIDS [16,33]. Due to a resulting dysfunction of the autonomic nervous system, SIDS victims have a diminished capacity to respond to physiological challenges during a vulnerable developmental period between 2 to 4 months after birth, when the majority of SIDS occurs [15,30,31]. Similar pathologic characteristics at autopsy have also been found among stillborn fetuses, suggesting that SIDS and unexplained stillbirth represent the same phenomenon [15-17]. SIDS and unexplained stillbirth share similar risk factors including male gender, seasonality, maternal smoking, a parity of 3 or more, race, extremes of maternal age, low education, single marital status and low socio-economic status [15,18,19]. Antepartum hypoxia is believed to be responsible for the majority of unexplained antepartum stillbirths [34]. Many complications of pregnancy that necessitate iatrogenic delivery involve fetal hypoxia [35]. The recent temporal increase in medically indicated deliveries has been shown to coincide with a reduction in stillbirth rates [36] and the decline in SIDS follows a similar temporal pattern.

Changes in environmental factors, such as the infant’s sleeping position, safe sleep environments and second hand smoke have changed over time, and likely contributed to a substantial fraction of the decline in gestational age-specific rates of SIDS. More recently, side-sleeping and bed-sharing have been identified as risk factors for SIDS [37]. Other explanations for the temporal reduction in SIDS rates have been proposed including temporal changes in SIDS case ascertainment and death certificate coding practices. Studies have shown that the reduction in SIDS in the United States paralleled a temporal increase in deaths caused by ‘accidental suffocation and strangulation’ [38]. However, the decline in SIDS has been observed in many countries and a similar shift in coding practices worldwide is unlikely [1,20,39]. For instance, a significant temporal reduction in SIDS was observed in the United Kingdom, where SIDS cases were carefully evaluated by the Confidential Enquiry into Stillbirths and Death in Infancy team. This included a thorough clinical and criminal investigation and a post-mortem examination by a pediatric pathologist [7].

Our study adds to the literature suggesting that unexplained fetal death and SIDS may have common causal pathways. The fetuses-at-risk approach shows that gestational age-specific rates of stillbirth and SIDS both increase with advancing gestation. Further, the fetuses-at-risk approach resolves the paradoxical relationship between gestational age-specific rates of SIDS and plurality and shows that twins are at higher risk of SIDS at all gestational ages. This adds to a growing body of evidence that suggests an intrauterine etiology for SIDS [15-17]. Our finding that changes in the gestational age distribution contributed significantly to the temporal reduction in SIDS rates also supports a common causal pathway between stillbirth and SIDS. The iatrogenic left shift in the gestational age distribution at term and post-term gestation that has occurred in recent decades has had a positive impact on both stillbirth and SIDS.

Our study has several limitations. We used death certificates to identify SIDS and this information is subject to potential misclassification as death certification processes are limited in their ability to incorporate all information about the circumstances leading to death [40,41]. A systematic misclassification, such as an increasing preference for other types of diagnoses than SIDS may have contributed to the temporal decline in gestational age-specific rates of SIDS in our study. The diagnostic code for SIDS changed from ICD-9 (code 798.0) to ICD-10 (code R95) in 2000, and SIDS cases were more likely to be reported under the new ICD-10 coding rules [42]. This change was estimated to artificially increase the SIDS rates by about 3% [42]. However, any effect of changes in coding practices would likely be uniform across all gestational ages. The relative reduction in SIDS in our study was larger among singletons compared with twins between 1995–96 vs 2004–05, whereas a similar relative reduction was observed for singletons and twins between 1995–96 and 2001–02. This is in contrast to data from England, where the relative SIDS reduction was larger in twins as compared with singletons [9]. Potential explanations for this discordance include differences in the time period when the largest changes in the gestational age distribution and in SIDS rates occurred in the 2 countries. Data from the United States show that the SIDS decline was larger among twins as compared with singletons from 1990 to 2005 (rate ratio 0.35, 95% CI: 0.28-0.43 among twins vs rate ratio 0.43, 95% CI: 0.41-0.45 among singletons). However, clinical estimates of gestational age were not available prior to 1995 and this restricted the time period of our study.

## Conclusions

In conclusion, our study indicates that in addition to the back-to-sleep campaign, temporal changes in the gestational age distribution may have contributed to the overall reduction in SIDS. This effect of a temporal shift towards earlier gestation at delivery has been observed predominantly at term and post-term gestation, and to a larger extent among twins. These findings support the hypothesis that antenatal factors contribute to the origins of SIDS, and endorse the concept of similar causal pathways between unexplained fetal death and SIDS.

## Abbreviations

SIDS, Sudden Infant Death Syndrome; NCHS, National Centre for Health Statistics; ICD, International Classification of Diseases; CI, confidence interval.

## Financial disclosure

None of the authors have a personal financial relationship relevant to this article.

## Competing of interests

The authors declare that they have no competing interests.

## Authors’ contributions

SL, JAH and KSJ contributed to the conception and design of the study, SL conducted the data analysis and drafted the manuscript and JAH and KSJ revised the manuscript for intellectual content; all authors approved the final version of the manuscript.

## Pre-publication history

The pre-publication history for this paper can be accessed here:

http://www.biomedcentral.com/1471-2393/12/59/prepub
